# Development and results of the German National Strategy for the Promotion of Breastfeeding: a participatory process

**DOI:** 10.3389/fpubh.2025.1555139

**Published:** 2025-09-11

**Authors:** Anna-Kristin Brettschneider, Jana Steindl, Beate Matthes-Bolz, Iris Lehmann, Jennifer Hilger-Kolb, Elena Roskosch, Gabriele Strauß, Mariell Hoffmann, Regina Ensenauer

**Affiliations:** ^1^Department of Child Nutrition, Max Rubner-Institut (MRI) - Federal Research Institute of Nutrition and Food, Karlsruhe, Germany; ^2^Press and Public Relation, Max Rubner-Institut (MRI) - Federal Research Institute of Nutrition and Food, Karlsruhe, Germany; ^3^EBS Universität für Wirtschaft und Recht, Oestrich-Winkel, Germany

**Keywords:** breastfeeding, national strategy, Germany, health promotion, public health nutrition, participatory process

## Abstract

Breast milk is the optimal nutrition for infants. However, the proportion of mothers who exclusively breastfeed their children in Germany is low (40% end of 4th month; 13% end of 6th month). A systematic evaluation of breastfeeding promotion has ranked Germany as moderately breastfeeding-friendly. Therefore, the federal government has commissioned the Department of Child Nutrition at the Max Rubner-Institut to coordinate the interdisciplinary development and implementation of a German National Strategy for the Promotion of Breastfeeding. In a participatory process, over 150 stakeholders have conceptualized measures to promote breastfeeding in seven strategic fields: (1) “Evidence-based guidelines,” (2) “Basic/advanced training and continued professional development,” (3) “Prevention and healthcare structures,” (4) “Breastfeeding promotion by municipalities,” (5) “Breastfeeding in the workplace,” (6) “Marketing of breast-milk substitutes,” and (7) “Systematic breastfeeding monitoring.” Measures include e.g., the development of an evidence-based medical guideline for breastfeeding duration and interventions to promote breastfeeding as a basis for education and training for involved occupational groups. Another measure is to establish a new research field at the Department of Child Nutrition to develop a systematic breastfeeding monitoring system. Additional measures concern the development of more breastfeeding-friendly framework conditions in healthcare structures, municipalities, and in the workplace, as well as regulations for the marketing of breast-milk substitutes and the development of communication strategies to promote breastfeeding. Following the adoption by the Federal Cabinet, the strategy serves as the basis for a sustainable improvement of breastfeeding promotion in Germany.

## 1 Introduction

Breast milk is the best nutrition for infants. Its composition is perfectly tailored to the requirements of the infant (1). In addition to the macro- and micronutrients required for healthy development, breast milk contains a large number of bioactive components, including immune factors, hormones, enzymes, pre- and probiotic substances and growth factors (2). There is widespread evidence that breastfeeding provides short- and long-term health benefits: breastfed children compared to non-breastfed children suffer less often from diarrhea, respiratory diseases (3), and acute otitis media in the first few years of life (4), have a lower risk of becoming overweight (5, 6) and less often develop type 1 and 2 diabetes mellitus (7, 8). Also, breastfeeding has positive effects for mothers, as it reduces the risk of several diseases like breast and ovarian cancer (9), type 2 diabetes mellitus (9, 10), and hypertension (10).

The World Health Organization (WHO) recommends “exclusive breastfeeding for the first 6 months of life and subsequent continued breastfeeding up to the age of 2 years or beyond” (11, 12). In Germany, the current recommendation on infant nutrition proposes “exclusive breastfeeding for the first 4 to 6 months followed by continued breastfeeding as long as mother and child wish” (13, 14).

However, despite the benefits of breastfeeding, in Germany the proportion of mothers who exclusively breastfeed their children is low: 40% of the children are breastfed exclusively up to the end of the fourth and 13% up to the end of the sixth month of life (15). In some neighboring countries such as Austria, Poland and Luxembourg, breastfeeding rates are even lower (4 months: 29–30.5%; 6 months: 1.9–3%), while in the Netherlands they are higher (4 months: 45%; 6 months: 39%) compared to Germany (16, 17). Regarding determinants of breastfeeding initiation, the level of maternal education is known to be a relevant factor: in comparison to 94.5% of mothers with a high educational level, only 68.5% of the mothers with a low educational level initiate breastfeeding (18). Further determinants related to a lower breastfeeding initiation rate include pre-conceptional overweight or obesity (19), multiple births, smoking during pregnancy, preterm delivery (18), and cesarean section (20).

Several approaches to promote breastfeeding have been initiated on an international and national level. Original concepts of breastfeeding promotion were outlined in the Innocenti Declaration on the protection, promotion and support of breastfeeding adopted in 1990 (21), the “Babyfriendly Hospital Initiative” (BFHI) of the WHO and the United Nations Children’s Fund (UNICEF) (22), and the International Code of Marketing of Breast-milk Substitutes of the WHO (23). Based on these initiatives, the “Global Strategy for the Nutrition of Infants and Young Children” of the WHO was adopted by the World Health Assembly (WHA) in 2002, which calls for changes in structures around the world in favor of breastfeeding (24). In its global nutrition goals, the WHO aims to increase the breastfeeding rate for exclusive breastfeeding in the first 6 months to over 50% by the year 2025 (25). An update from 2023 shows that the rate is already 48% (26), which represents an increase of 10% compared to 10 years earlier (27). However, the number of countries included differs between both surveys, e.g., in this most recent estimation, the number of included countries from the WHO European Region was markedly lower than in the earlier data.

In 1994, the German Federal Government joined the Innocenti Declaration and founded the German National Breastfeeding Committee, whose main task is to promote breastfeeding in Germany. Since 2019, the German National Breastfeeding Committee has been located at the Department of Child Nutrition at the Max Rubner-Institut (MRI) (28). In Germany, breastfeeding promotion is also anchored in the national health targets “Grow up healthy: life competence, physical activity, nutrition” and “Health all around childbirth” of the Federal Ministry of Health (29).

However, despite the large number of different approaches to promote breastfeeding in Germany, breastfeeding rates remain low. Therefore, the international research project “Becoming Breastfeeding Friendly” (BBF) (30, 31) was initiated by the Federal Ministry of Food and Agriculture (BMEL) as a systematic evaluation of breastfeeding promotion in Germany (32). From 2017 to 2019, BBF was conducted by the Healthy Start – Young Family Network (33) at the Federal Center of Nutrition (BZfE) and the German National Breastfeeding Committee in cooperation with the Yale School of Public Health, who had designed the project. They identified a BBF Index score of 1.7 (range 0–3, with 0 as lowest and 3 as highest), indicating that Germany is moderately breastfeeding-friendly. For comparison, UK conducted the BBF process at around the same time, identifying a BBF Index score of 1.1 for England and Wales, which is also considered moderately breastfeeding-friendly (34, 35). Scotland reached a BBF Index score of 2.4, representing a ‘strong scaling up environment’ (36).

Given the strengths and needs identified in the German BBF process, calls to action for scaling up breastfeeding promotion were derived by the BBF group. They identified the need for developing a German National Strategy for the Promotion of Breastfeeding (37) as a key recommendation, which was picked up and followed by the BMEL.

The Department of Child Nutrition at the MRI is responsible for coordinating the development and implementation of this strategy. The overall aim of the German National Strategy for the Promotion of Breastfeeding is to improve the conditions for breastfeeding in Germany in order to increase the proportion of breastfed children and to promote the short-term and long-term health of children and mothers. Breastfeeding promotion comprises coordinated efforts to increase breastfeeding initiation and duration through public awareness, policy advocacy, professional training, and supportive environments. To conceptualize the strategy, the MRI carried out a participatory, interdisciplinary process, in which a large number of stakeholders relevant to the field of breastfeeding promotion were closely involved. The aim of this manuscript was to outline the participatory process of developing the German National Strategy for the Promotion of Breastfeeding and to present the results relating to the aims and measures derived from this process.

## 2 Methods

**Figure 1:**
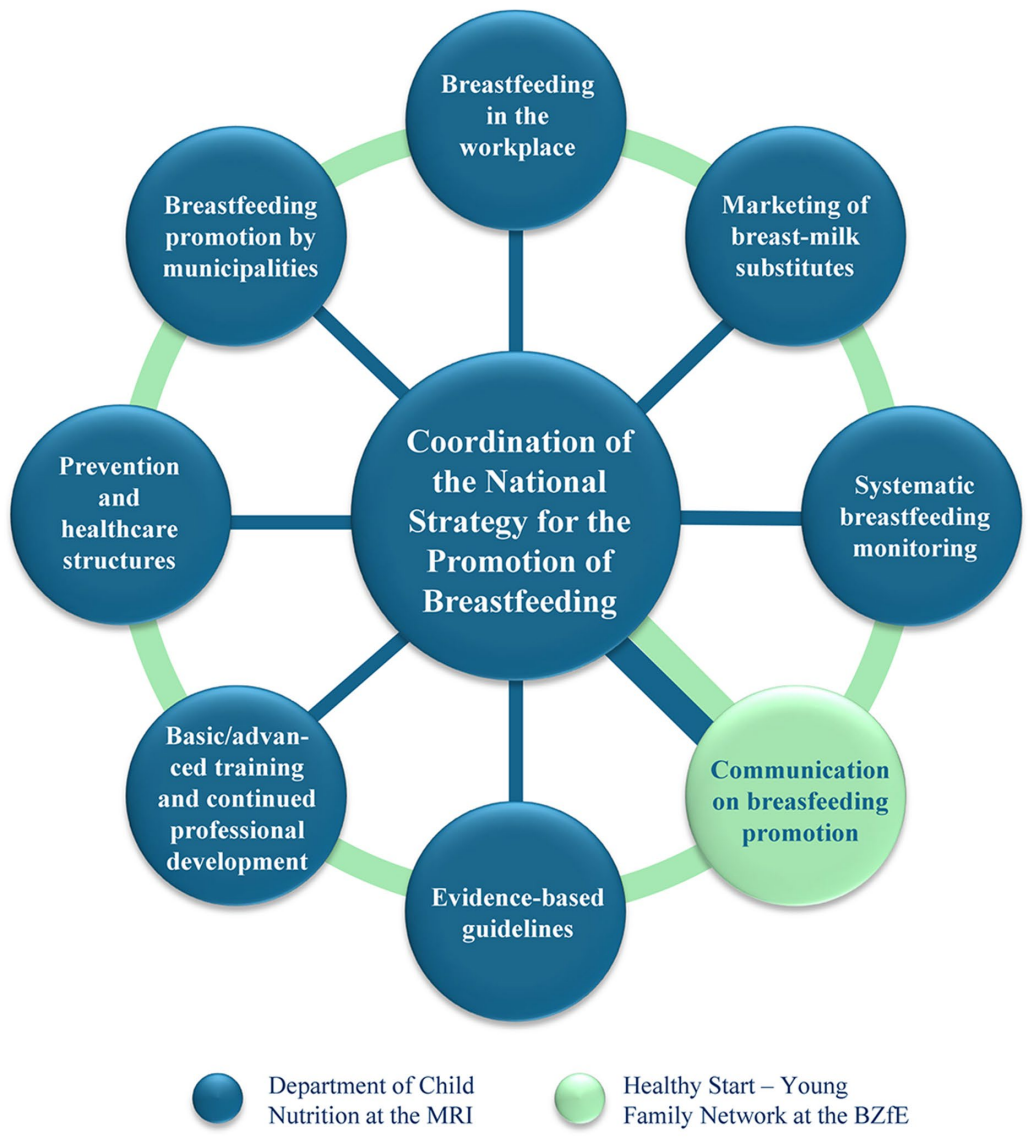
Strategic fields of the German National Strategy for the Promotion of Breastfeeding. MRI, Max Rubner-Institut; BZfE, Federal Center of Nutrition.

As part of the German BBF process, the Breastfeeding Gear Model was used to identify relevant fields of action to promote breastfeeding (30, 37). Based on these results, the main areas of action to promote breastfeeding were summarized in seven strategic fields: (1) “Evidence-based guidelines,” (2) “Basic/advanced training and continued professional development,” (3) “Prevention and healthcare structures,” (4) “Breastfeeding promotion by municipalities,” (5) “Breastfeeding in the workplace,” (6) “Marketing of breast-milk substitutes,” and (7) “Systematic breastfeeding monitoring” (Figure 1). Communication on breastfeeding promotion is a cross-sectional task that is linked to the seven strategic fields. In parallel, the strategy is aligned with the five action areas of the Ottawa Charter for Health Promotion, aiming to create supportive environments, develop personal skills, reorient health services, strengthen health policy, and foster community action (38, 39). Since greater public involvement seems to achieve greater acceptance among experts, stakeholders and the public, a participatory process for the development of the new national breastfeeding strategy on a broad societal basis was chosen (38–41). Within the strategic fields, the participatory process defined aims and measures on how to improve the conditions for breastfeeding in Germany in a sustainable manner.

**Figure 2:**
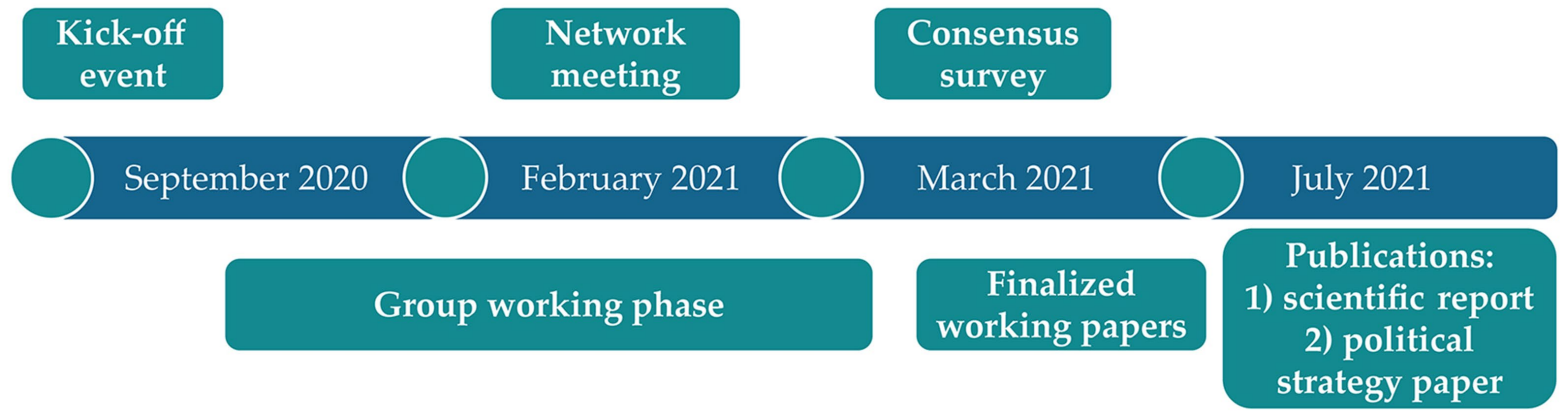
Timeline and steps of the participatory process for the development of the German National Strategy for the Promotion of Breastfeeding.

Figure 2 displays the timeline and the steps of developing the German National Strategy for the Promotion of Breastfeeding which took place from September 2020 until July 2021 including an online kick-off event, a group working phase, a meeting to promote networking between strategic fields (network meeting), a consensus survey, and finalization of the working papers.

The coordination team for developing the German National Strategy for the Promotion of Breastfeeding consisted of staff from both the Department of Child Nutrition and the Press and Public Relation section at the MRI. They coordinated the conceptualization of aims and measures of the seven strategic fields (Figure 1) by involving stakeholders and experts with various backgrounds (policy, pediatrics, midwifery etc.). Involving stakeholders with direct contact to the target group enabled the integration of the respective perspectives, i. e., from pregnant women, mothers, (young) families and their environment. The experts and stakeholders in each strategic field formed the so-called Strategic Groups.

In the beginning, the coordination team conducted an online kick-off event for the development of the strategy, in which over 150 stakeholders in the field of breastfeeding promotion joined and participated, the so-called NaSt Group (Supplementary “Members of the NaSt Group”). The BBF project had identified numerous stakeholders (37) across various fields of action and involved some of them. The proposed stakeholders were considered in the development of the German National Strategy for the Promotion of Breastfeeding, and other relevant stakeholders were added in the course of the development process. The BMEL invited these stakeholders via e-mail to the kick-off event, encouraging their participation in strategy development. The NaSt Group comprised representatives from professional associations, healthcare provision and advocacy, public institutions, authorities, scientific institutions and universities, and non-governmental organizations (NGOs) and initiatives (Supplementary Table 1) as well as members of the German National Breastfeeding Committee, who took part in the entire process.

During the kick-off event, the Strategic Groups for each individual strategic field were established. All stakeholders were informed about the strategic fields in advance and had the opportunity to assign themselves to a strategic field of their choice and the associated Strategic Group depending on their interests or work focus. Some stakeholders also participated in two Strategic Groups. As participation in the strategic fields was a voluntary activity, which was also time-consuming due to the meetings and writing of the working papers including the measures, it was not possible for the stakeholders to participate in all Strategic Groups. Each Strategic Group was led by up to three expert group leaders who guided the group in terms of content and thus enabled a structured working process. The Strategic Group leaders were selected in advance by the coordination team based on their expertise. Within the kick-off event, each Strategic Group identified and prioritized aims to be achieved in the respective strategic field. Furthermore, the group members discussed the need for inclusion of additional stakeholders, disciplines and/or institutions for the development and subsequent implementation of processes to maximize expertise.

In the subsequent group working phase, each of the seven Strategic Groups came together in digital meetings to develop concrete measures for achieving the identified aims. The results of these meetings were documented in working papers. A digital network meeting with all the Strategic Group leaders was conducted in order to promote networking and clarify content-related overlaps between the Strategic Groups. After finalizing the working papers, a consensus survey was designed and conducted by the coordination team among all members of each Strategic Group. In the survey, consensus on the measures developed in the corresponding strategic fields was granted in order to identify minority votes within the Strategic Groups. The survey comprised tables with the measures developed in the working papers from the strategic fields (42) and was sent by e-mail to the respective group members. Members of the Strategic Groups were able to approve the measures by ticking the boxes in a table to indicate whether they agreed with the respective measure. They had the opportunity to make essential comments where necessary. Data were analyzed at the MRI.

Subsequently, two parallel processes were carried out: first, a scientific report (42) on the development of the German National Strategy for the Promotion of Breastfeeding was prepared, which contained background information, the participatory process, the complete working papers of the Strategic Groups and suggestions for implementation of the strategy. Second, a political strategy paper - the German National Strategy for the Promotion of Breastfeeding (43) was drafted, based on the results of the participatory process, and discussed with the BMEL following the involvement of the Federal Ministry of Health and the Federal Ministry for Family Affairs, Senior Citizens, Women and Youth. Both the scientific report (42) and the political strategy paper (43) were published in 2021, without having undergone independent scientific peer review.

As it plays an important role in all strategic fields, communication relating to breastfeeding promotion was considered a cross-sectional task. The Healthy Start – Young Family Network at the BZfE coordinated the development of a communication strategy for promoting breastfeeding together with experts in this field.

## 3 Priority topics

For each strategic field, measures have been derived to reach the respective aims and to improve the conditions for breastfeeding in a sustainable way. In the following, measures of the political strategy paper are described. An overview of aims and measures in the individual strategic fields is provided in Supplementary Table 2.

### 3.1 Strategic field “Evidence-based guidelines”

Breastfeeding promotion and breastfeeding counseling must be evidence-based. Thus, it is necessary to collect and evaluate current evidence-based literature to derive recommendations in medical guidelines for physicians and medical professionals and the population. The Working Group of Scientific Medical Societies e. V. (AWMF) is the German umbrella organization of medical societies and coordinates the development of medical guidelines. To date, there is no evidence-based AWMF guideline on breastfeeding duration and interventions to promote and support breastfeeding in Germany. However, breastfeeding is addressed in various other medical guidelines (e.g., the guideline for allergy prevention), providing in part inconsistent recommendations.

The aim of this strategic field is to ensure that all occupational groups who are in contact with pregnant or breastfeeding women provide uniform evidence-based recommendations on the duration of breastfeeding and breastfeeding promotion.

*Measures*:

1. Development of the AWMF-S3 guideline “Breastfeeding duration and interventions to promote breastfeeding” as the scientific basis for all breastfeeding promotion activities.
2. Integration of recommendations derived from the new guideline into revisions of other medical guidelines that include consistent recommendations and/or statements on breastfeeding.
3. Development of a report on the guideline in a comprehensible language and understandable for laypersons, e.g., (expectant) parents and families.
4. Dissemination of the guideline recommendations to professionals and the public.

### 3.2 Strategic field “Basic and advanced training and continued professional development”

The medical care that mothers receive during pregnancy, delivery and in the first 2 years of their child’s life makes a significant contribution to the initiation and duration of breastfeeding (44). In order to enable tailored care, the relevant professional groups need to have a broad knowledge and sufficient skills in breastfeeding promotion in accordance with their job-specific tasks and competencies. These professional groups are mainly physicians (especially pediatricians and gynecologists), midwives and nursing staff. Therefore, breastfeeding must be anchored and strengthened in the curricula for both basic and advanced training.

Aim of this strategic field is to provide all relevant occupational groups with the latest and evidence-based knowledge and skills in the field of breastfeeding and breastfeeding promotion and support through their basic and advanced training and continued professional development.

*Measures*:

1. Analysis of the existing curricula for both basic and advanced training and continued professional development of relevant occupational groups to identify a need for adaptation of contents.
2. Development of evidence-based learning contents based on the future AWMF-S3 guideline “Breastfeeding duration and interventions to promote breastfeeding” for all relevant professional groups and educational formats.

The different requirements and connecting points for educational content must be taken into account depending on the respective professional group (Table 1).

**Table 1:** Needs of individual occupational groups to be considered when developing evidence-based learning content.

Occupational group	Needs of occupations
Physicians	Strengthening of breastfeeding knowledge in medical education and corresponding advanced professional trainings (particularly obstetrics, pediatrics, general and internal medicine).
Midwives	Integration of results from research and breastfeeding-promoting projects in basic/advanced training of midwives.
Nurses	Specification and implementation of teaching contents in basic training and continued professional development.

### 3.3 Strategic field “Prevention and healthcare structures”

To prevent breastfeeding difficulties and to recognize and treat them at an early stage, it requires appropriate framework conditions and healthcare structures during pregnancy and after delivery. These should provide low-threshold breastfeeding counseling that considers both evidence-based literature and individual needs for all women.

The aim of this strategic field is to align the prevention and healthcare structures with (individual) needs and to provide appropriate framework conditions for breastfeeding promotion and breastfeeding counseling.

*Measures*:

1. Evaluation of the existing healthcare structures and consideration of whether the provided breastfeeding counseling services by health insurers and municipalities are sufficient.
2. If deficits are found, causes must be analyzed and suggestions should be drawn up on how these gaps can be addressed.
3. Application of the “Ten Steps to Successful Breastfeeding” (22) in all maternity clinics with outpatient units for pregnant women, pediatric clinics with premature and neonatal units, and birthing centers.

### 3.4 Strategic field “Breastfeeding promotion by municipalities”

The living environment has a major influence on breastfeeding behavior. In the area of breastfeeding promotion, low-threshold measures at the municipal level have the potential to reach even those women who start breastfeeding less often or stop earlier such as women with a low educational level compared to other groups. Many municipalities have recognized the relevance of promoting breastfeeding and strive to create a supportive environment for families. To be effective, these activities must be communicated to the public, and all stakeholders on site must be connected to build a network. The municipalities play a crucial role in fostering the social acceptance of breastfeeding.

The aim of this strategic field is to support families with needs-based, networked and low-threshold offers within the municipalities to promote breastfeeding.

*Measures*:

1. Setting up breastfeeding promotion as part of health promotion and prevention and linking to other preventive activities. Inclusion of breastfeeding promotion offers in municipal health planning and reporting, and support of stakeholders in the area of breastfeeding in establishing and expanding networks.
2. Development of the guideline “Breastfeeding-friendly municipality,” in order to promote public acceptance and improve the general conditions of breastfeeding in everyday life.
3. Establishing low-threshold offers for breastfeeding support, especially for vulnerable groups through municipal stakeholders as well as dedicated (infant) guidance systems in maternity clinics. For example, promotion of breastfeeding-friendly places and self-help activities such as breastfeeding and baby cafés help women to experience breastfeeding in practice and build networks.
4. Providing quality-approved information and materials on breastfeeding promotion by the federal government in order to support the municipalities in communication and public relations work.

### 3.5 Strategic field “Breastfeeding in the workplace”

To enable breastfeeding for all mothers who return to work, school, or university (shortly) after delivery or maternity leave, breastfeeding promotion in the workplace and in educational and training environments is essential. The German Maternity Protection Act (Mutterschutzgesetz-MuSchG) governs women’s health protection, but beyond the legal requirements, it is important for employers to create or improve adequate structural conditions that support and promote breastfeeding in workplaces and (educational) facilities.

Aim of this strategic field is to make workplaces as well as educational and training environments more breastfeeding-friendly.

*Measures*:

1. Encouragement of workplaces to implement measures for breastfeeding promotion. Corresponding information on the implementation of becoming breastfeeding-friendly can be compiled in a guidance document including clarification on the added value of breastfeeding friendliness for workplaces and employees.
2. Development of tailored information for pregnant and breastfeeding women, internal stakeholders and external multipliers to ensure that all stakeholders involved in breastfeeding promotion in the workplace are informed about rights, opportunities and duties.
3. Identification and dissemination of best practice examples to provide workplaces suggestions on how to implement measures to promote breastfeeding. Workplaces with exemplary breastfeeding promotion measures can also act as role models by offering a “Breastfeeding Fellowship” to interested workplaces.
4. Investigation of the need for action to improve the general conditions for female students, pupils and family members providing support, women not covered by statutory insurance schemes and (solo) self-employed workers.
5. Establishing a network between those involved in breastfeeding promotion in the workplace, as well as between scientists who perform research in this area (at national level and, where appropriate, at international level) to make knowledge more available and to encourage implementation, taking all relevant interest groups into account.

### 3.6 Strategic field “Marketing of breast-milk substitutes”

Advertising often suggests that breast milk can easily be replaced by industrial infant formula and causes uncertainty in parents (45). Therefore, there are legal regulations for the marketing of breast-milk substitute products (46). The non-legally binding “International Code of Marketing of Breast-milk Substitutes” of the WHO (23) was taken into account when the regulations were drawn up.

The aim of this strategic field is to bring the regulations on the marketing of breast-milk substitutes more into the focus of experts, authorities and the public in order to limit the influence of industry corporations.

*Measures*:

1. Comprehensible communication of regulations for the marketing of breast-milk substitutes with information materials for professionals and the general public.
2. Identification of possible needs for further regulations, e.g., regarding implementation of additional recommendations of the WHO Code. Furthermore, complementary approaches for implementation beyond existing standards will be identified, such as self-regulation by industry.
3. Raising the awareness of the supervisory authorities of the German federal states, who are responsible for monitoring compliance with legal regulations for the marketing of breast-milk substitutes. Evaluating if a central reporting office for violations against the regulations is necessary.
4. Sensitization to the problem of industry influence and the resulting conflicts of interest among those involved. For example, information and advertising materials must be handled critically, and it must be examined to what extent advanced professional trainings can be carried out for relevant occupational groups (e.g., physicians) without conflicts of interest from the speakers and without funding and involvement from industry.

### 3.7 Strategic field “Systematic breastfeeding monitoring”

Systematic acquisition of data on breastfeeding is crucial to quantify the accuracy of measures to promote breastfeeding. However, a population-wide data collection system has not been established in Germany to date.

The aim of this strategic field is to establish a system for the acquisition of breastfeeding data that continuously provides population-wide data.

*Measures*:

1. Establishing a research area for the development and implementation of a systematic breastfeeding monitoring system at the Department of Child Nutrition at the MRI.
2. Identifying and establishing appropriate instruments. To obtain data on rates of breastfeeding initiation, the focus is on the mandatory quality assurance procedures in perinatal medicine, which covers all births in hospitals. A second approach is to collect information about breastfeeding behavior during the regular well-child visits, in order to ascertain data on breastfeeding duration.
3. Inclusion of data from existing studies to analyze determinants of breastfeeding behavior.
4. Development of a concept for data storage and data analysis.
5. Regular publication of the continuously collected data and results.

### 3.8 Cross-sectional task “Communication on breastfeeding promotion”

Communication allows the imparting of knowledge to the population, families, and all stakeholders, to shape perceptions, and to change attitudes or even behavior. As communicative measures have also been derived from the other seven strategic fields, the Healthy Start – Young Family Network at the BZfE will consider these measures when developing the communication concept for the National Strategy for the Promotion of Breastfeeding.

The aims of this cross-sectional task are to consolidate and enhance communication activities regarding breastfeeding, to increase social acceptance and promote a breastfeeding-friendly atmosphere, as well as to inform and support families from pregnancy to delivery and breastfeeding with target-group specific information.

*Measures (examples)*:

1. Continuous expansion of target-group specific evidence-based information and materials with applied content. For example, print media for families with clear messages, which present breastfeeding in a realistic, practical and solution-oriented manner.
2. Dissemination of information among various target groups through adapted press and media work.
3. Implementation of measures with a coordinated content, design and editorial approach across different channels and statements by trustworthy personalities from politics, sport and media to increase social acceptance and promote a breastfeeding-friendly atmosphere.

## 4 Consensus survey

The consensus survey was carried out for all measures included in the scientific report that relate to the strategic fields “Evidence-based guidelines,” “Basic/advanced training and continued professional development,” “Prevention and healthcare structures,” “Breastfeeding promotion by municipalities,” “Breastfeeding in the workplace,” and “Marketing of breast-milk substitutes.” The summarized results are shown in Table 2. Survey response rates were between 27 and 53%, and the mean response rate across all strategic fields was 42.5%.

**Table 2:** Results of the consensus survey addressing the agreement of stakeholders with the measures of the strategic fields.

Strategic field^1^	Stakeholders (n)^2^	Response rate	Measures (n)	Consent to measures (range)	Consent to measures (mean)
Evidence-based guidelines	25	52%	10	85–100%	95%
Basic/advanced training and continued professional development	40	53%	25	86–100%	97%
Prevention and healthcare structures	32	41%	21	62–100%	85%
Breastfeeding promotion by municipalities	38	39%	16	80–100%	92%
Breastfeeding in the workplace	15	27%	11	75–100%	89%
Marketing of breast-milk substitutes	21	43%	19	44–100%	75%

1 The consensus survey did not include the measures of the strategic field “Systematic breastfeeding monitoring” and the cross-sectional task “Communication on breastfeeding promotion” (see 4).

2 Total *n* = 171 as multiple participation was possible through involvement in two strategic fields.

The survey asked for consensus for each measure of a strategic field, showing a high level of support for the results achieved through the participatory process, except for eight measures in the strategic field “Marketing of breast-milk substitutes” and three measures in the strategic field “Prevention and healthcare structures.” Mean consent for the measures in the strategic fields ranged from 85 and 97%, with the exception of the measures in the strategic field “Marketing of breast-milk substitutes” (75%). As there were disagreements in the strategic field “Systematic breastfeeding monitoring” at the beginning of the working phase regarding the structure of the working paper, a proposal for a new structure of the working paper and individual measures were already agreed upon in advance, and therefore no further consensus survey was conducted. The cross-sectional task “Communication on breastfeeding promotion” was not part of the participatory process coordinated by the MRI, so it was not included in the consensus survey.

## 5 Discussion

The German National Strategy for the Promotion of Breastfeeding provides the basis for sustainably improving and further developing breastfeeding promotion in Germany. Through a participatory process, involving a large number of relevant stakeholders, a variety of measures and concepts contributed to the development of this strategy for making Germany more breastfeeding-friendly in the short-, medium- and long-term.

Participatory research is also emphasized in the Ottawa Charter of the WHO as a fundamental principle for the practice of health promotion and is based on the assumption that projects are more effective and sustainable when the people addressed are actively included in the change process (39). In the public health sector, participatory methods are often used, for example, in the context of intervention programs, research projects, or evaluations and include mostly the involvement of citizens or people addressed (47–50). Using a participatory approach within the development of the German National Strategy for the Promotion of Breastfeeding integrated interdisciplinary perspectives. The process has also led to knowledge transfer and networking of the relevant stakeholders, which might promote the development of future supporting structures.

During the strategy development, the expert group leaders were invited to a digital network meeting to foster personal networking, discuss content-related overlaps between different strategic fields, and make agreements on how to deal with them. The results of the meeting aided in revising the working papers, leading to the inclusion of relevant information on networking between the strategic fields in the final scientific report. Overarching measures between the strategic fields “Prevention and healthcare structures” and “Breastfeeding promotion by municipalities” include developing healthcare structures that facilitate easier implementation by municipalities. The strategic field “Evidence-based guidelines” will be networked with all other strategic fields, as the new guideline will serve as the basis for future breastfeeding promotion activities. The cross-sectional task “Communication on breastfeeding promotion” is linked to every strategic field for disseminating the recommendations of the new guideline or developing target group-specific information materials in the fields of “Breastfeeding promotion by municipalities” and “Breastfeeding in the workplace.”

With the working papers derived from the Strategic Groups, the basis for the German National Strategy for the Promotion of Breastfeeding has been built. The development of aims, essential measures to achieve these aims, and ways of implementation are an important gain in knowledge and thus a major part of the process. Through the participatory process, different levels of knowledge within the Strategic Groups have been shared. This shared knowledgebase enabled a qualified evaluation of the measures by the stakeholders in the discourse. The focus was not on the consensus of all of those involved in designing the measures, but rather on the process of exploring all possibilities and determining the extent of the consensus within the Strategic Group. The consensus survey showed a high agreement for most measures among members of the Strategic Groups, which confirms that the working papers are not based on individual opinions. By conducting the consensus survey, the participatory process of the development of the German National Strategy for the Promotion of Breastfeeding was completed, and the strategy was adopted in the Federal Cabinet in July 2021.

The implementation of the measures of the seven strategic fields is coordinated by the Department of Child Nutrition at the MRI. The cross-sectional task “Communication on breastfeeding promotion” is coordinated by the Healthy Start – Young Family Network at the BZfE.

Prioritization strengthens the basis for sustainable and effective breastfeeding promotion. Since the new guideline “Breastfeeding duration and interventions to promote breastfeeding” (see 3.1) is the scientific basis for further breastfeeding promotion activities, this step is prioritized and is ongoing. Furthermore, education and training should be strengthened by integrating current, scientifically based breastfeeding promotion topics. Establishing continuous, population-wide breastfeeding monitoring is also a priority, as a sound database is required to manage and evaluate the measures. Implementation of further measures in the strategic fields “Evidence-based guidelines” and “Systematic breastfeeding monitoring” is being conducted by the Department of Child Nutrition at the MRI. In addition, the Healthy Start – Young Family Network has already published first communicative measures e.g., a communication guide for all aspects of breastfeeding that offers criteria for stigma-sensitive communication and specific examples of text and visual language (51).

### 5.1 Strength and limitations

One strength is that due to the large number of stakeholders involved, very different expertise and perspectives have been considered. The developed strategy strongly refers to practical experience and thus is likely to be more sustainable. By being closely involved in the development, the stakeholders may identify themselves with the strategy and thus support the measures and take over responsibilities during future implementation work.

In addition, minority views were considered, which was motivating for participation on the one hand, but on the other hand, it might be a limiting factor, since it can also include measures that may not be of major relevance. However, not all measures suggested by the stakeholders were adopted within the political paper of the German National Strategy for the Promotion of Breastfeeding. Another limiting factor is that due to the large number of participants, a wide range of measures have been developed. Since not all measures can be implemented at the same time, prioritization is necessary. Due to the COVID-19 pandemic it was necessary to change the participation concept from a personal to a virtual process with all its challenges and advantages. The digital kick-off event offered special opportunities when carried out virtually e.g., a high participation rate, but also limited some work steps. The consensus survey showed a low response rate. A reason might be that the willingness to participate was quite high at the beginning due to the online process, but during the development phase, the proportion of active group members decreased. A further limitation is that the measures were specifically adapted to the situation in Germany and thus may not be easily transferable to other countries.

## 6 Conclusion

By adopting the strategy in the Federal Cabinet, the Federal Government shows its commitment and its will to support breastfeeding promotion on a national level. Since the German National Strategy for the Promotion of Breastfeeding addresses measures in the short-, medium- and long-term, the implementation will take several years. Besides the government, many other stakeholders, above all the health and economic authorities in the federal states, the joint self-administration in the health system, the universities, the professional associations, and experts, will be involved to implement the strategy. Also, the stakeholders who have worked in the strategic fields are invited to support the implementation of the strategy in future steps. An advisory committee will support the implementation and aims at prioritizing goals and measures and evaluating the processes. By developing a system of breastfeeding monitoring, changes in breastfeeding behavior through the implementation of the German National Strategy for the Promotion of Breastfeeding will become visible. Re-evaluation of the status of breastfeeding promotion in Germany should be considered in order to determine further needs for adjustments.
