# Supplementary material for: Development and results of the German National Strategy for the Promotion of Breastfeeding: a participatory process
Source: Front Public Health. 2025 Sep 11;13:1555139. doi: 10.3389/fpubh.2025.1555139 (PMC12460087; doi:10.3389/fpubh.2025.1555139)
Supplement: Supplementary file 1 [file Table_1.docx]

Supplementary Material

# Supplementary Information

Members of the NaSt Group

All stakeholders involved in the participatory process of developing the German National Strategy for the Promotion of Breastfeeding who consented to the publication of their names in the scientific report (1):

Prof. Dr. Michael Abou-Dakn, PD Dr. Ute Alexy, Lisa Apini-Welcland, Isabel Auer, Juliane Beck, Dr. Monika Berns, Anja Bier, Dr. Brigitte Borrmann, Iris-Susanne Brandt-Schenk, Dr. Evelyn Breitweg-Lehmann, Dr. Anna-Kristin Brettschneider, Dr. Christine Bruni, Elisabeth Burghardt, Sandra Deissmann, Dr. Julia Dienst, Nicole Dirks-Wetschky, Tatjana Drewitz, Prof. Dr. Florian Ebner, Dr. Stefanie Eiser, Miriam Elsaeßer, Olivia Engel, Prof. Dr. Regina Ensenauer, Sonja Eppler, Dr. Judit Etspüler, Maria Flothkötter, Dörte Freisburger, Ulrika Gehrke, Katja Gilbert, Dr. Alexandra Glaß, Dr. Antje Gottberg, Prof. Dr. Melita Grieshop, Susanne Großkopf, Dr. Susanne Grylka, Prof. Dr. Matthias Hastall, Sandra Heintz, Petra Hemmerle, Kathrin Herold, Vera Hesels, Jennifer Hilger-Kolb, Dr. Evelyn Jantscher-Krenn, Simone Martina Hock, PD Dr. Sandra Hummel, Nikola Jakobs, Dr. Anne Just, Dr. Thomas Kauth, Gudrun Kinzel, Dr. Melanie Klein, Ingrid Kloster, Kirsten Knuth, Karin Kriwanek, Dr. Ilona Krois, Katharina Krüger, Prof. Dr. Alfred Längler, PD Dr. Anja Lange, Christina Law-McLean, Dr. Burkhard Lawrenz, Simone Lehwald, Prof. Dr. Lars Libuda, Anja Lohmeier, Dr. Stephanie Lücke, Karolina Luegmair, Kerstin Marx, Karin Maucher, Prof. Dr. Eva Mildenberger, Dr. Gunda Morales, Alexandra Müller-Helm, Barbara Müllerschön-Göhring, Dr. Uta Nennstiel, Aline Okantah, Christel Opitz-Lüders, Norbert Pahne, Dr. Wolfgang Panter, Andrea Paucke, Mechthild Paul, Kerstin Plack, Silke Raab, Monika Radke, Rita Rausch, Lysann Redeker, Utta Reich-Schottky, Dr. Petra Reihl, Ilona Renner, Dr. Maren Reyer, Dr. Stefanie Rosin, Elien Rouw, Kateryna Savina, Gudrun Schaarschmidt, Dr. Michael Scheele, Prof. Dr. Viviane Scherenberg, Dr. Anja Schienkiewitz, Nicole Schlaeger, PD Dr. Dietmar Schlembach, Dorothee Schmitz, Annett Schmok, Sabine Scholz-de Wall, Dr. Susen Schulze, Petra Schwaiger, PD Dr. Erika Sievers, Dr. Skadi Springer, Dr. Jens Stupin, Nicole Tempel, Dr. Sybill Thomas, Dr. Claudia Thräne-Pietruk, Dr. Gabriele Trost-Brinkhues, Birgit Unger, Martina van der Weem, Aleyd von Gartzen, Susanne Warmbrunn-Koerth, Stephanie Wilhelm

# Supplementary Tables

**Supplementary Table 1.** Examples of professional associations, actors in healthcare provision and advocacy, public institutions and authorities, scientific institutions and universities, non-governmental organizations and initiatives involved in developing the strategy, represented by stakeholders.

| **Representatives of…** |  |
| --- | --- |
| Professional associations | e. g. Professional Association of Pediatricians e. V. (German: Berufsverband der Kinder- und Jugendärzt*innen e. V. (BVKJ)), German Midwives Association e. V. (German: Deutscher Hebammenverband e. V. (DHV)), The German Nutrition Society (German: Deutsche Gesellschaft für Ernährung e. V. (DGE)) |
| Actors in healthcare provision and advocacy | e. g. Federal Joint Committee (German: Gemeinsamer Bundesausschuss (G-BA)),  National Association of Statutory Health Insurance Physicians (German: Kassenärztliche Bundesvereinigung (KBV)), health insurances, National Association of Statutory Health Insurance Funds (German: GKV-Spitzenverband) |
| Public institutions, authorities | e. g. Healthy Start Network (Netzwerk Gesund ins Leben) at the Federal Center of Nutrition (BZfE),  German Federal Centre for Health Education (German: Bundeszentrale für gesundheitliche Aufklärung (BZgA)), National Centre for Early Prevention (German: Nationale Zentrum Frühe Hilfen (NZFH)), Consumer centers (German: Verbraucherzentralen), The Federal Office of Consumer Protection and Food Safety (German: Bundesamt für Verbraucherschutz und Lebensmittelsicherheit (BVL)) |
| Scientific institutions and universities | e. g. Robert Koch Institute (German: Robert Koch-Institut (RKI)),  Institute of Diabetes Research at Helmholtz Munich (German: Institut für Diabetesforschung (IDF), Helmholtz Zentrum München), Charité – Universitätsmedizin Berlin,  Protestant University of Applied Sciences Berlin (German: Evangelische Hochschule Berlin (EHB)) |
| Non-governmental organizations and initiatives | e. g. La Leche Liga e. V., Association supporting the WHO/UNICEF Baby-Friendly Initiative (BFHI) e. V. (German: Verein zur Unterstützung der WHO/ UNICEF- Initiative "Babyfreundlich" (BFHI) e. V.), Human Milk Bank Initiative (German: Frauenmilchbank Initiative (FMBI) e. V.) |

**Supplementary Table 2.** Overview table of aims and measures of the German National Strategy for the Promotion of Breastfeeding (political strategy paper) in the individual strategic fields.

|  | **Strategic fields** | | | | | | |
| --- | --- | --- | --- | --- | --- | --- | --- |
|  | **Evidence-based guidelines** | **Basic/advanced training and continued professional development** | **Prevention and healthcare structures** | **Breastfeeding promotion by municipalities** | **Breastfeeding in the workplace** | **Marketing of breast-milk substitutes** | **Systematic breastfeeding monitoring** |
| **Aims** | - Ensure all professionals in contact with pregnant or breastfeeding women give consistent, evidence-based advice on breastfeeding duration and promotion | - Provide the latest and evidence-based knowledge and skills in the field of breastfeeding and breastfeeding promotion | - Align prevention and healthcare structures with individual needs  - Provide suitable conditions for breastfeeding promotion and counseling | - Support families with needs-based, networked and low-threshold offers within the municipalities to promote breastfeeding | - Make workplaces and educational / training environments more breastfeeding-friendly | - Raise awareness of breast-milk substitute marketing regulations among experts, authorities, and the public  - Limit influence of industry corporations | - Establish a system for continuous, population-wide breastfeeding data collection |
| **Measures** | Guideline development „Breastfeeding duration and interventions to promote breastfeeding“ | Curriculum analysis of educational formats for relevant occupational groups | Evaluation of existing healthcare structures | Integration of breastfeeding promotion into health initiatives and municipal planning | Encourage work-places for breastfeeding promotion | Comprehensible communication of regulations for the marketing of breast-milk substitutes | Creating a research area for the development and implementation of a systematic breastfeeding monitoring system |

Supplementary Table 2. (cont.)

| **Measures** | Integration of guideline recommendations to other medical guidelines | Development of evidence-based learning content for all relevant occupational groups and edu-cational formats | Address gaps in the existing healthcare structures | Development of a "Breastfeeding-friendly municipality" guideline | Development of target group specific information about rights, opportunities and duties | Identification of possible needs for further regulations | Identifying and establishing appropriate instruments |
| --- | --- | --- | --- | --- | --- | --- | --- |
|  | Guideline development for laypersons |  | Application of the „Ten Steps to Successful Breastfeeding“ in in maternity clinics | Establishment of low-threshold breastfeeding support services | Identification and dissemination of best practice examples | Raising the awareness of the supervisory authorities of the German federal states | Inclusion of data from existing studies |
|  | Dissemination of the guideline recommendations |  |  | Provide quality-approved breastfeeding information and materials by the federal government | Investigation of need for action to improve the general conditions for female students, pupils and family members providing support, women not covered by statutory insurance schemes and (solo) self-employed workers | Sensitization for the problem of influence by the industry and the resulting conflicts of interest of those involved | Development of a concept for data storage and data analyses |
|  |  |  |  |  | Establishing a network between those involved in breastfeeding promotion in the workplace |  | Regular publication of results |

**References**

1. Brettschneider AK, Steindl J, Matthes B, Ensenauer R. Nationale Strategie zur Stillförderung – Ergebnisse des partizipativen Prozesses: Max Rubner-Institut (MRI) (2021) [10.01.2025]. Available from: <https://www.mri.bund.de/de/stillstrategie/>.
